# Use of Avacopan in Patients With Antineutrophil Cytoplasmic Antibody-Associated Vasculitis and Estimated Glomerular Filtration Rate <15 ml/min per 1.73 m^2^

**DOI:** 10.1016/j.ekir.2024.01.006

**Published:** 2024-01-11

**Authors:** Bryce Barr, Kim Cheema, Aurore Fifi-Mah, Stephanie Garner, Louis-Philippe Girard

**Affiliations:** 1 Department of Medicine, Rady Faculty of Health Sciences, University of Manitoba, Winnipeg, Manitoba, Canada; 2 Department of Medicine, Cumming School of Medicine, University of Calgary, Calgary, Alberta, Canada

**Keywords:** avacopan, glomerulonephritis, vasculitis

## Introduction

Rapidly progressive glomerulonephritis represents a severe manifestation of antineutrophil cytoplasmic antibody-associated vasculitis (AAV), and is associated with substantial morbidity and mortality.^1^ Kidney function following 6 months of therapy is predictive of future requirement of kidney replacement therapy, and patients who require kidney replacement therapy have the worst long-term outcomes.^1^ Therefore, maximal recovery of kidney function is imperative to improve survival and quality of life.

**Figure 1:**
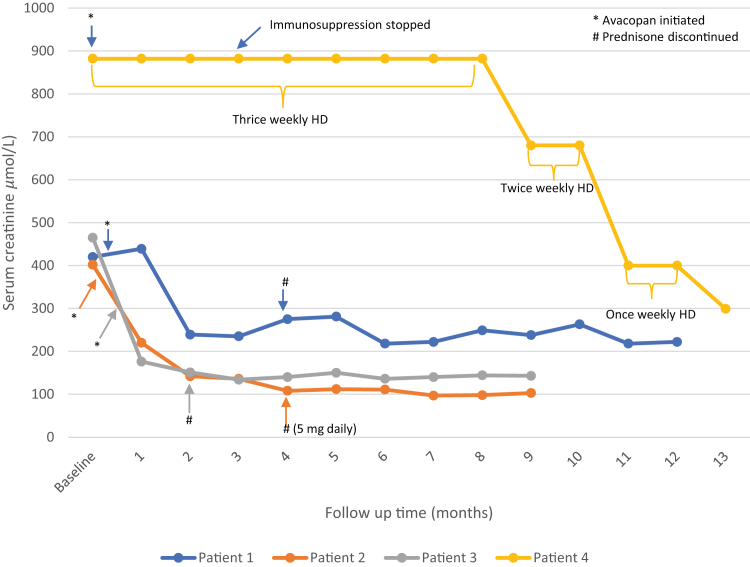
Clinical course of patients treated with avacopan.∗Avacopan initiated. #Prednisone discontinued

The ADVOCATE trial demonstrated the superior efficacy of avacopan, a small molecule C5a receptor antagonist, over prednisone with respect to a primary outcome of sustained remission at 52 weeks, with reduced glucocorticoid toxicity in patients with AAV.^2^ Avacopan resulted in a significant increase in estimated glomerular filtration rate (eGFR), including the subset of patients with eGFR <20 ml/min per 1.73 m^2^.^3^ The ADVOCATE trial excluded patients with eGFR <15 ml/min per 1.73m^2^; data in this subgroup are currently limited to 1 case series in which all patients required kidney replacement therapy.^4^ Here, we report the experience using avacopan at our center in 4 patients with AAV with baseline eGFR <15 ml/min per 1.73 m^2^. Their baseline characteristics and clinical course are summarized in Table 1 and Figure 1.

**Table 1:** Characteristics and outcomes of patients treated with avacopan

Characteristics	Patient 1	Patient 2	Patient 3	Patient 4
Age	76	59	80	63
Sex	F	F	F	M
Follow-up (mo)	12	9	9	13
ANCA type	MPO	PR3	MPO	MPO
Berden Class	Mixed	Mixed	Focal	Mixed
Brix Score	9	3	3	11
Creatinine^1^	420	402	465	882
eGFR	8	10	7	5
Urine PCR	128.8	103	N/A	114
ANCA titer (AI)	>800	561	48.1	4.3
Hematuria at time zero (/hpf)	>30	>30	150	21–30
Induction therapy	CYC	CYC/RTX	CYC	RTX
PLEX (Y/N)	N	N	N	N
Day avacopan initiated	19	14	21	28
Remission	Yes	Yes	Yes	Yes
Creatinine at follow-up^a^	222	103	143	299
eGFR at follow-up^b^	18	51	32	18
Urine PCR at follow-up^c^	58.8	34.3	3.97	40
ANCA titer at follow-up (AI)	584.2	2.8	26.3	4.3
Hematuria at follow-up (/hpf)	0–2	0	3–5	0–2
Cumulative oral GC	1720	1055	2160	1190
Liver enzyme abnormalities	None	None	None	None
Serious infection^d^	No	No	No	No
Maintenance therapy	RTX	RTX	prednisone 5mg/d	None

ANCA, antineutrophil cytoplasmic antibody; CYC, cyclophosphamide; eGFR, estimated glomerular filtration rate; F, female; GC, glucocorticoid; hpf, high-power field; M, male; MPO, myeloperoxidase; PCR, protein-to-creatinine ratio; PLEX, plasma exchange; PR3, proteinase 3; RTX, rituximab.

a Units are μmol/l.

b Units are ml/min per 1.73 m^2^.

c Units are mg/mmol.

d Defined as infection requiring hospitalization.

## Case Descriptions

### Patient 1

A 76-year-old woman with history of hypertension, dyslipidemia, and stage G3b chronic kidney disease (baseline creatinine 145 μmol/l), presented to hospital with epistaxis and malaise. Her creatinine was found to be 420 μmol/l (eGFR 9 ml/min per 1.73 m^2^), with urinalysis demonstrating hematuria and proteinuria. Myeloperoxidase antibody titer was found to be >800 AI. Kidney biopsy was performed, which demonstrated 27% cellular crescents, 46% global glomerulosclerosis, and 20% to 25% normal glomeruli with 25% to 50% interstitial fibrosis and tubular atrophy. She was treated with methylprednisolone 500 mg i.v. daily for 3 days followed by prednisone 60 mg orally (PO) daily with tapering per the reduced-dose PEXIVAS regimen, in addition to cyclophosphamide 7.5 mg/kg i.v. as per the European Vasculitis Study Group dosing schedule for 6 doses.^5^ Two weeks after discharge, she was seen in outpatient follow-up, where serum creatinine remained 438 μmol/l. To minimize glucocorticoid exposure and attempt to recover kidney function, she initiated avacopan 30 mg PO twice daily. Prednisone was reduced to 20 mg PO daily and tapered by 5 mg per week until discontinuation. At month 6, maintenance therapy with rituximab was initiated. At month 12, she remained off prednisone and in clinical remission with creatinine 222 μmol/l (eGFR 18 ml/min per 1.73 m^2^).

### Patient 2

A 59-year-old previously healthy woman presented with otalgia and scleritis and was found to have a creatinine of 402 μmol/l (eGFR 10 ml/min per 1.73 m^2^) from a baseline of 60 μmol/l, 9 months earlier. Urinalysis demonstrated hematuria, and proteinase 3 antibody was positive with titer 561 AI. Kidney biopsy revealed 33% cellular crescents, no global glomerulosclerosis, and 41% normal glomeruli with 10% to 25% interstitial fibrosis and tubular atrophy. She was treated with methylprednisolone 500 mg i.v. daily for 3 days followed by prednisone 60 mg PO daily with tapering per the reduced-dose PEXIVAS regimen, in addition to combination i.v. cyclophosphamide 12.5 mg/kg ×2 doses and rituximab 1g ×2 doses. Two weeks after the initiation of cyclophosphamide, given the severity of her kidney disease, she initiated avacopan 30 mg PO twice daily, with prednisone tapered from 30 mg daily to discontinuation over 4 weeks. At month 6, rituximab was initiated for maintenance therapy. At month 9, she remained off prednisone and in clinical remission with creatinine 103 μmol/l (eGFR 51 ml/min per 1.73 m^2^).

### Patient 3

An 80-year-old woman with history of hypertension, dyslipidemia, and chronic kidney disease G3a A1 (baseline creatinine 91 μmol/l) presented to hospital with cough, otalgia, and malaise. She was found to have a creatinine of 465 μmol/l (eGFR 8 ml/min per 1.73 m^2^) with an active urine sediment along with a myeloperoxidase at a titer of 48.1 AI, which prompted a kidney biopsy. Biopsy demonstrated 15% glomerular necrosis, 25% global glomerulosclerosis, severe necrotizing arteritis, and 10% to 25% interstitial fibrosis and tubular atrophy. She was treated with methylprednisolone 500 mg i.v. daily for 3 days followed by prednisone 50 mg PO daily with tapering per the low-dose PEXIVAS regimen, in addition to cyclophosphamide 7.5 mg/kg i.v. (European Vasculitis Study Group dosing schedule) for 6 doses.^5^ She was discharged on day 14. Given the severity of her kidney disease, she initiated avacopan 30 mg PO twice daily on day 21. Following this, her care was transferred to another nephrologist. No maintenance therapy was prescribed; the reason for this was not clear. At month 9, she remained in clinical remission on prednisone 5 mg daily, with creatinine 143 μmol/l (eGFR 32 ml/min per 1.73 m^2^).

### Patient 4

A 63-year-old man with recently diagnosed hypertension presented to hospital with 3 weeks of fatigue and malaise. His antecedent blood work 7 years earlier demonstrated creatinine 80 μmol/l. On presentation, he was found to have a creatinine of 882 μmol/l with active urinalysis, peripheral neuropathy, and computed tomography of the chest showing pulmonary nodules. Testing for antineutrophil cytoplasmic antibody revealed myeloperoxidase of 4.3 AI; and a kidney biopsy demonstrated 23% fibrocellular crescents, and 49% global glomerulosclerosis with 80% interstitial fibrosis and tubular atrophy. He was treated with methylprednisolone 500 mg i.v. daily for 3 days followed by prednisone 30 mg daily for the first 4 weeks of treatment. In addition, he received rituximab 1g i.v. ×2, and was started on hemodialysis on the fourth day of admission. Following 4 weeks of therapy and continued requirement for dialysis, he initiated avacopan 30 mg PO twice daily, and prednisone was tapered by 5 mg per week until discontinuation. At his 3-month follow-up appointment, he remained dialysis-dependent and immunosuppression, including avacopan, was discontinued. Ten months following diagnosis, despite no maintenance immunosuppression, he began to show signs of kidney function recovery. At month 13, he was dialysis-independent on no immunosuppression, with serum creatinine 299 μmol/l, (eGFR 20 ml/min per 1.73 m^2^).

## Discussion

Patients with eGFR <15 ml/min per 1.73 m^2^ were excluded from the ADVOCATE trial; there are minimal data on the use of avacopan in this population. In our cohort, no serious adverse events, including infection were noted, and glucocorticoids were tapered rapidly following initiation of avacopan. Avacopan will be used for 12 months in all patients, except patient 4 who discontinued at 3 months. All patients experienced substantial eGFR recovery despite profound kidney dysfunction at presentation.

Patients with AAV with eGFR <15 ml/min per 1.73 m^2^ are at the highest risk for kidney failure and mortality.^1^ The mechanisms by which avacopan may lead to improvement of eGFR are unclear. It is possible that the addition of avacopan induces more rapid remission, as evidenced by lower urine albumin-to-creatinine ratio in the avacopan group at week 4 in the ADVOCATE trial,^2^ with more rapid remission limiting histologic damage. This remains speculative in the absence of repeated biopsy studies comparing the impact of avacopan versus prednisone on kidney histology. In the cohort from ADVOCATE with eGFR <20 ml/min per 1.73 m^2^, more than 80% of patients had myeloperoxidase-antineutrophil cytoplasmic antibody subtype,^3^ which is associated with more glomerulosclerosis, fibrosis, and worse long-term kidney function as compared to proteinase 3-antineutrophil cytoplasmic antibody.^6^ Therefore, mitigation of fibrosis with avacopan may explain superior eGFR recovery in this subgroup.

Our report has limitations. First, our cohort is limited to 4 patients with no control group. Second, none of the patients in our cohort were treated with plasma exchange; therefore, data regarding the combination of avacopan with plasma exchange in those with severe kidney failure remain limited. Finally, although our results are encouraging, it is important to recognize that 1 of our patients continued to require dialysis at follow-up. Therefore, it remains important to have realistic expectations about the degree of kidney function recovery expected with avacopan. Furthermore, this case highlights the need for early identification of disease, and the early institution of treatment to maximize the improvement in eGFR and limit disease-associated damage.

In conclusion, we present 4 cases describing the use of avacopan in individuals with AAV and eGFR <15 ml/min per 1.73 m^2^ at presentation. Avacopan appeared to be safe, reduced glucocorticoid exposure, and resulted in substantial eGFR recovery in 4 individuals. More study is required to understand the short-term and long-term impacts of avacopan on kidney function in this subgroup of patients with AAV.

## Disclosure

BB reports consultancy agreements with Otsuka Canada, Novartis, and GlaxoSmithKline. KC reports honoraria from Alexion and Otsuka Canada, consultancy agreements with Alexion and Novartis, and advisory board membership for AB002 Drug and Safety Monitoring Board. AF-M reports consultancy agreements with Otsuka Canada. SG reports consultancy agreements with UCB, Abbvie, Novartis, Janssen, Sanofi, and Otsuka Canada. LG reports honoraria from Otsuka Canada and honoraria from Otsuka Canada, as well as being site Principal Investigator for the ADVOCATE and PEXIVAS trials, and a CanVasc core member.

## Patient Consent

The authors declare that they have obtained consent from the patients discussed in the report.
